# Thomas Keith and Ovariotomy

**Published:** 1880-09

**Authors:** 


### THOMAS KEITH AND OVARIOTOMY.

In the *American Journal of Obstetrics* for April of the present year, Dr. Sims contributes an interesting and instructive sketch of the most successful ovariotomist of the world, with descriptions of his methods of operation, and a detailed account of the proceedings in one case. The method of Dr. Keith's working becomes of special interest, not only to those interested in ovariotomy, but to every one engaged in serious surgical procedures; his success from the first has been far better than that of any other ovariotomist, and lately he has had seventy-three successive cases of recovery. In short in his hands the operation suggested by John Hunter in 1786, pronounced cruel and outrageous in 1824, has become so admirably systematized, that recovery is the rule, and death as the result, a rarity. Dr. Sims after carefully analyzing the question, and no one is better able to do so, concludes that the amazing success is due first and chiefly, to the patient and prolonged care in the complete arrest of intra-peritoneal hemorrhage, tying one point after another with carbolized cat-gut ligatures, sponging and exploring with the utmost accuracy of scrutiny, ligating seemingly unimportant little oozing points "until he feels sure that there is not a point left unsecured from which bleeding, after the establishment of reaction, could possibly take place." This completed, the depths of the pelvic cavity are cleansed with small sponges, until they come out clean. The pedicle is treated with either ligature or cautery. When the latter is used, Keith does not allow the iron to retain even a red heat; but dips it into cold water until the redness has disappeared, and with the black hot iron slowly severs the tissues, afterwards smoothing them off with another iron at a brown heat. The drainage tube is used if there be an effusion of *bloody serum*. Dr. Sims concludes from his observations, that bloody serum is more dangerous in the peritoneal cavity than either pure blood or pure serum; consequently, if there be no adhesions, even though ascites exist, drainage is unnecessary; if doubt arises as to the perfect control of hemorrhage, drainage is advisable. The antiseptic method of Lister is minutely employed; and to this measure is Keith indebted for raising his percentage from eighty-seven to ninety-three of recoveries.

Before closing the external wound, Keith adopts the plan of Spencer Wells, of placing a broad flat sponge, (first wrung out in hot carbolized water), over the intestines, passing his sutures over this; after the sutures are *in situ* they are seized at their centre and drawn, one-half upwards, one-half downwards, thus allowing space for the sponge to be withdrawn; this sponge receives any drops of blood that occur from the passage of the suture needle, and also shows if any other bleeding has happened; in case there is evidence of hemorrhage, another search for bleeding points is instituted. Dr. Sims has added a second sponge, which is clasped in lock forceps and passed into the pelvis, so that no bleeding can arise while the last steps of the operation are in progess, that will not be declared before the wound is finally closed.

After-treatment, since Listerism has been used, is of the simplest. If pain occurs, twenty drops of laudanum, or its equivalent, is given by the rectum, to be repeated if necessary. For the first forty-eight hours the patient gets no nourishment, except a little brandy and ice as they may be required. At the end of this time light nourishment, as beef-tea and milk; in a day or two, unless some contra-indication exists, the bowels are moved by an enema, and, if all goes well, a more nutritious diet is allowed.—*St. Louis Courier of Medicine.*

